# Teaching Palliative Care to Emergency Medicine Residents Using Gamified Deliberate Practice-Based Simulation: Palliative Gaming Simulation Study

**DOI:** 10.2196/43710

**Published:** 2023-08-16

**Authors:** Jessica Stanich, Kharmene Sunga, Caitlin Loprinzi-Brauer, Alexander Ginsburg, Cory Ingram, Fernanda Bellolio, Daniel Cabrera

**Affiliations:** 1 Department of Emergency Medicine Mayo Clinic Rochester, MN United States; 2 Division of Palliative, Department of Medicine Mayo Clinic Rochester, MN United States; 3 Department of Health Science Research, Division of Health Care Policy and Research Mayo Clinic Rochester, MN United States

**Keywords:** palliative care, emergency medicine, gaming simulation, resident education, medical education, residency, end of life, palliative, dying, death, interpersonal skill

## Abstract

**Background:**

Emergency departments (EDs) care for many patients nearing the end of life with advanced serious illnesses. Simulation training offers an opportunity to teach physicians the interpersonal skills required to manage end-of-life care.

**Objective:**

We hypothesized a gaming simulation of an imminently dying patient using the LIVE. DIE. REPEAT (LDR) format, would be perceived as an effective method to teach end-of-life communication and palliative care management skills.

**Methods:**

This was a gaming simulation replicating the experience of caring for a dying patient with advanced serious illness in the ED. The scenario involved a patient with pancreatic cancer presenting with sepsis and respiratory distress, with a previously established goal of comfort care. The gaming simulation game was divided into 4 stages, and at each level, learners were tasked with completing 1 critical action. The gaming simulation was designed using the LDR serious game scheme in which learners are allowed infinite opportunities to progress through defined stages depicting a single patient scenario. If learners successfully complete the predetermined critical actions of each stage, the game is *paused*, and there is a debriefing to reinforce knowledge or skills before progressing to the next stage of the gaming simulation. Conversely, if learners do not achieve the critical actions, the game is *over*, and learners undergo debriefing before repeating the failed stage with an immediate transition into the next. We used the Simulation Effectiveness Tool–Modified survey to evaluate perceived effectiveness in teaching end-of-life management.

**Results:**

Eighty percent (16/20) of residents completed the Simulation Effectiveness Tool–Modified survey, and nearly 100% (20/20) either strongly or somewhat agreed that the gaming simulation improved their skills and confidence at the end of life in the following dimensions: (1) better prepared to respond to changes in condition, (2) more confident in assessment skills, (3) teaching patients, (4) reporting to the health care team, (5) empowered to make clinical decisions, and (6) able to prioritize care and interventions. All residents felt the debriefing contributed to learning and provided opportunities to self-reflect. All strongly or somewhat agree that they felt better prepared to respond to changes in the patient’s condition, had a better understanding of pathophysiology, were more confident on their assessment skills, and had a better understanding of the medications and therapies after the gaming simulation. A total of 88% (14/16) of them feel more empowered to make clinical decisions. After completing the gaming simulation, 88% (14/16) of residents strongly agreed that they would feel more confident communicating with a patient and prioritizing care interventions in this context.

**Conclusions:**

This palliative gaming simulation using the LDR format was perceived by resident physicians to improve confidence in end-of-life communication and palliative care management.

## Introduction

Emergency departments (EDs) are facing growing numbers of patients with advanced serious illnesses. Palliative care interventions in the ED capture high-risk patients at a time of crisis and can change the course of the disease, improving patient-centered outcomes [1]. These patients require skillful communication so that clinicians can tailor care to the patient’s values, goals, and preferences [2,3]. Emergency physicians receive minimal palliative and end-of-life education training during residency, and palliative and end-of-life care training within emergency medicine (EM) has been identified as an area of need [4-6]. A survey of Canadian emergency medicine programs found that 38.5% have palliative and end-of-life care curricula, mostly in the form of lectures. Barriers to implementing palliative care curricula were lack of time (84.6%) and curriculum development concerns (80.8%) [4]. To fill this gap, different programs have been created to expand communication skills training to EM providers [7,8]. Some programs have used simulation to empathically deliver serious news and discuss goals of care through role-playing and small group learning [9]. A significant change in practice has not been achieved, despite this training, which empowered our team to create a novel gaming simulation method.

In this paper, we describe a novel gamified deliberate-practice simulation module targeting palliative management concepts and communication in an imminently dying patient using the previously described gaming simulation method: LIVE. DIE. REPEAT (LDR) [10]. The LDR framework uses a serious game scheme where learners are allowed infinite lives to progress through multiple stages depicting a single patient scenario. A serious game is an educational tool focused on problem-solving and learning while borrowing from the entertaining constructs of a video game [11]. The learner faces a discrete simulated clinical situation (a level or stage), where success is defined by achieving predetermined critical actions within a specified time frame. If learners complete the expected objectives, the game is *paused*. This allows for focused debriefing, providing an educational foundation and reinforcing correct knowledge, skills, and attitudes. The game resumes, and learners can advance to the next level. Conversely, if at the end of a level learners are unsuccessful at completing critical actions, the game is *over*, and learners must undergo a targeted debriefing before gameplay is resumed to first repeat the failed stage before and then immediately continuing to the next level. The short debriefings are intended to provide performance feedback and offer an immediate opportunity to apply the concepts learned during the debriefing. This integrates the idea of deliberate practice, an established method to achieve superior performance through recognition of defined measurement standards, rote experience, analysis of behaviors, and repetition of skills [10,12]. A critical aspect of the LDR framework is the usage of Kolb experiential model loops intertwined between the levels and the debriefing [13]. The learners can execute a concrete action, then have a period of reflection, conceptualize the new knowledge, and then experiment again with the newly acquired set of skills.

We aimed to evaluate the EM residents’ perception of the use of the LDR gaming simulation in teaching and building confidence in managing care at the end of life in a time-constrained environment. The didactic gaming simulation aimed to expose learners to critical concepts in end-of-life care, including review of advance care planning (ACP) documentation and appropriate interpretation, how to efficiently conduct an informed goals-of-care discussion, and managing the actively dying patient.

## Methods

### Overview

This is an observational study of gaming simulation encounters. We adhere to the Simulation-Based Research Extensions for the STROBE (Strengthening the Reporting of Observational Studies in Epidemiology) Statements for reporting [14].

### Intervention

The LDR palliative scenario was developed by 3 board-certified EM physicians. A board-certified palliative medicine physician also participated in the gaming simulation during debriefings. The scenario involved a simulated patient with stage 4 pancreatic cancer and an automatic implantable cardioverter defibrillator (AICD), presenting with hypoxemic respiratory failure and sepsis secondary to pneumonia, with a previously established goal for comfort care. Learners also interacted with an actor playing the role of the patient’s daughter who acts as a care partner and advocate for the patient.

The simulation game was divided into 4 stages, and at each level, learners were tasked with completing 1 critical action. Prior to the start of level 1 the residents were prebriefed by one of the EM faculty simulation facilitators regarding the LDR game format. This prebriefing took place in a classroom outside of the clinical simulation room. Residents rotated through each stage in teams. Learners not currently participating in each level could view the scenario via video feed to observe its content and progression, allowing them to participate in debriefing and future levels of the game. The debriefing took place in the same classroom location.

**Figure 1:**
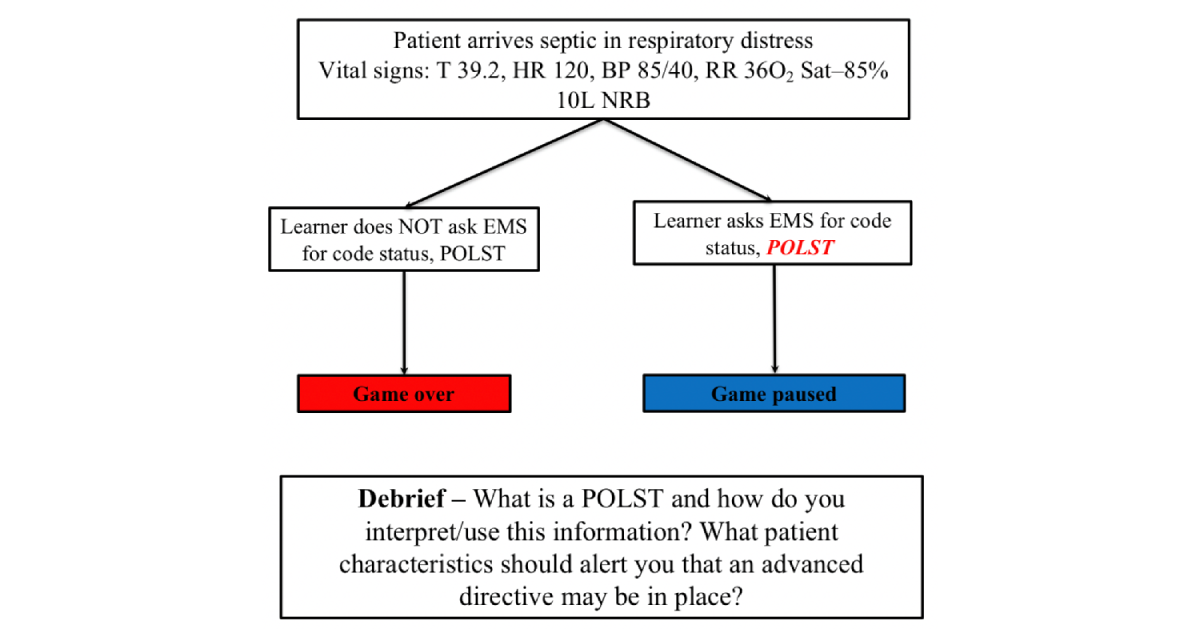
Level 1—Critical action: ask for POLST. BP: blood pressure; EMS: emergency medical services; HR: heart rate; NRB: non-rebreather; POLST: provider order for life-sustaining treatment; RR: respiratory rate.

Level 1 begins with an embedded participant portraying the role of an emergency medical services (EMS) provider arriving at the hospital with a patient in severe respiratory distress. The EMS provider shares that the patient has stage 4 pancreatic cancer and called for shortness of breath. The patient arrives persistently hypoxic despite the use of oxygen by a nonrebreather. Persistent and declining oxygen saturations are used during the gaming simulation to apply pressure on the learner to decide how to proceed with care. The critical action to succeed at this level and pause the game is for the learner to ask for a provider order for life-sustaining treatment (POLST) form (Figure 1). If the resident learner does not ask for a POLST and indicates that he or she will prepare to intubate the patient, this is deemed as a failed critical action (game *over*). During the debrief of level 1, faculty introduced the concept of ACP documents, how they can apply to care for an imminently ill patient, when they are completed, and by whom. The ACP documents discussed included advance directives, POLST forms, and ACP notes specific to the medical record used at the Mayo Clinic. After debriefing, learners who did not achieve the critical action return to the simulation gameplay at the beginning of level 1. Those who successfully accomplish level 1 critical actions start at level 2.

**Figure 2:**
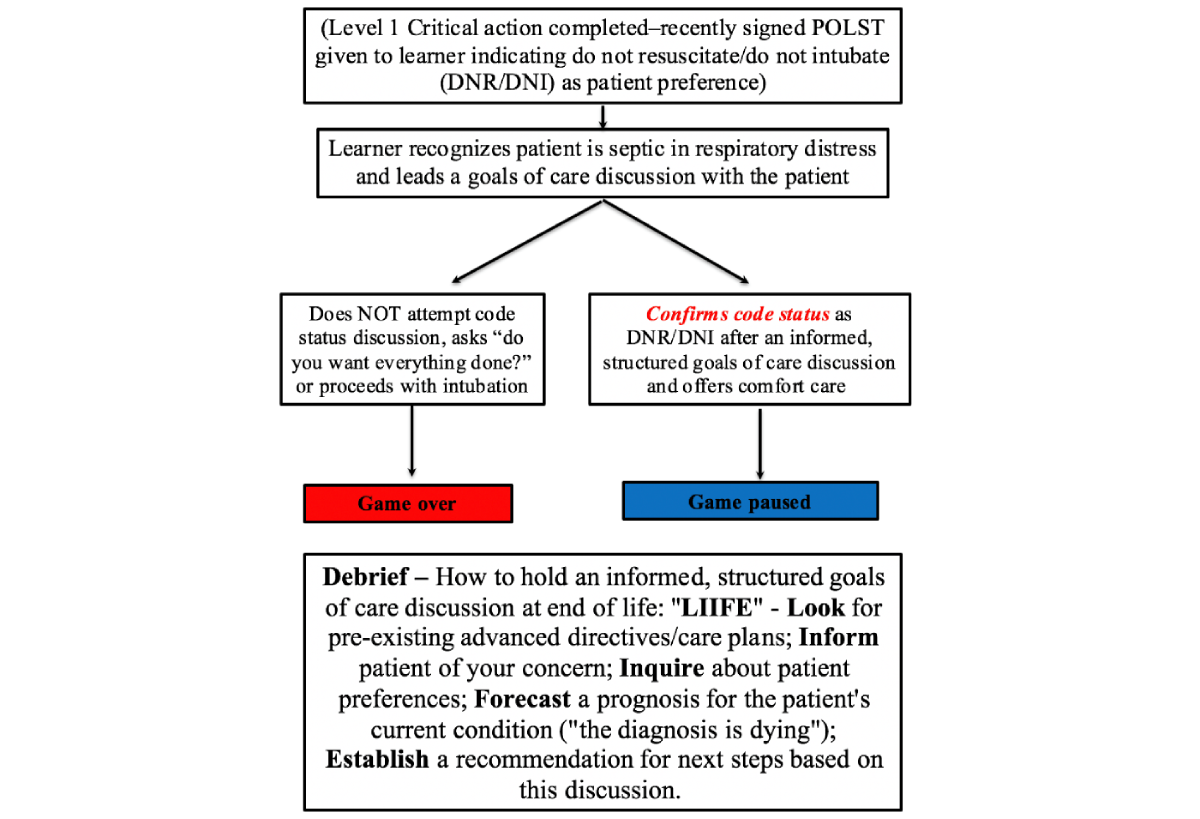
Level 2—Critical action: goals of care confirmation. DNI: do not intubate; DNR: do not resuscitate; POLST: provider order for life-sustaining treatment.

Level 2 begins after the POLST document is obtained, and EM providers are made aware of the patient’s prior wishes for comfort-focused care (Figure 2). The critical action for level 2 is for learners to interpret the patient’s ACP documentation within the clinical context and address the patient’s current goals of care. The standardized patient is portrayed in extremis; however, the patient demonstrates the capacity to make decisions. If the learning team frames the discussion surrounding intubation as providing care versus withholding care (“do you want everything done?”), the level is failed (game *over*) and learners must repeat this stage. If, however, the patient’s prior goals of care are confirmed and comfort-focused care is framed as an active intervention, the level is considered successfully achieved (game *pause*). During the debrief, a structured approach to a goals-of-care discussion was outlined. The acronym “LIIFE” was used to provide a framework to permit a more structured approach to the goals-of-care discussion. LIIFE stands for: *Look* for ACP documents; *Inform* the patient they are very sick, and the provider is worried they are dying; *Inquire* about the patient's functional status and current quality of life; *Forecast* a prognosis for the patient’s current condition; and *Establish* a recommendation for the next steps based on the discussion. This acronym was designed by one of the EM faculty facilitators and was created using a variety of resources including expert experience and other published studies [9,15-17].

**Figure 3:**
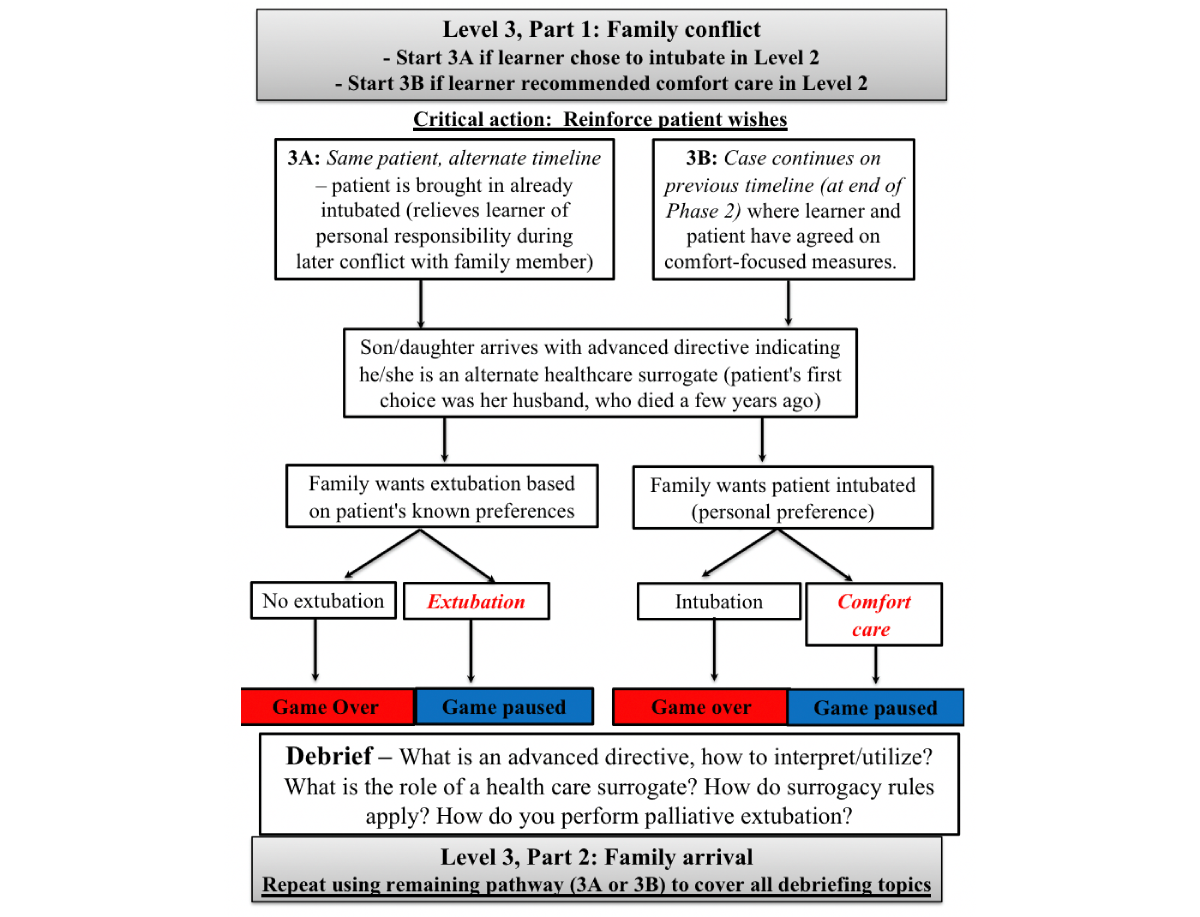
Level 3—Family conflict critical action: reinforce patient wishes.

Level 3 continues the clinical encounter at the point after which POLST documentation has been obtained and interpreted, and after the learner has discussed with the patient the next steps in care. Level 3 involves a family conflict and has 2 potential pathways (3A and 3B) depending on the decisions made during level 2 (Figure 3). However, both level 3A and 3B pathways are ultimately played during the course of the game so that all educational objectives are met.

- If the game was *paused* in level 2 (ie, comfort-focused care was confirmed), the game resumes in level 3B where the patient remains on a nonbreather, tachypneic with increased work of breathing. The patient’s daughter (a live actor) arrives demanding the patient be intubated due to her level of breathing distress and reports that she is the power of attorney based on the patient’s advance directive, which is provided to the resident in this stage. The game is*paused* if the care plan of comfort-focused care is reiterated with the daughter. The level is considered failed (game *over*) if the learners move toward intubating the patient.
- If, however, level 2 ended in game *over* (intubation was chosen), the game continues in level 3A—which resets the entire scenario and presents a contrasting situation. This stage begins when the patient arrives at the hospital already having been intubated by EMS due to persistent hypoxia and altered mental status. The patient’s daughter (a live actor) arrives irate that the patient is intubated, given her familiarity with the patient’s POLST documentation and demands the “tube be removed,” emphasizing once again her position as the patient’s power of attorney on the provided advance directive documents. The relative change of the clinical situation (patient arrives already intubated) allows the learner to concentrate on a discussion where there is a clear mismatch between the patient’s goals and the intervention performed but without personal responsibility for the intubation itself. Learners must move toward extubating the patient in order for the game to be paused.

Once both levels 3A and 3B are completed, the entirety of the level 3 debriefing includes education on patient capacity and using the patient’s POLST as a guide when considering self-determination in the context of conflicting health care surrogate wishes. The debriefing also includes the incorporation of prognostic awareness in providing firm recommendations for care rather than relegating the decision to patients or family and how to provide reassurance that the symptom of dyspnea can be treated without intubation while acknowledging this could result in the patient dying. Education on extubating a patient and the medications used to provide comfort at the end of life were also discussed.

**Figure 4:**
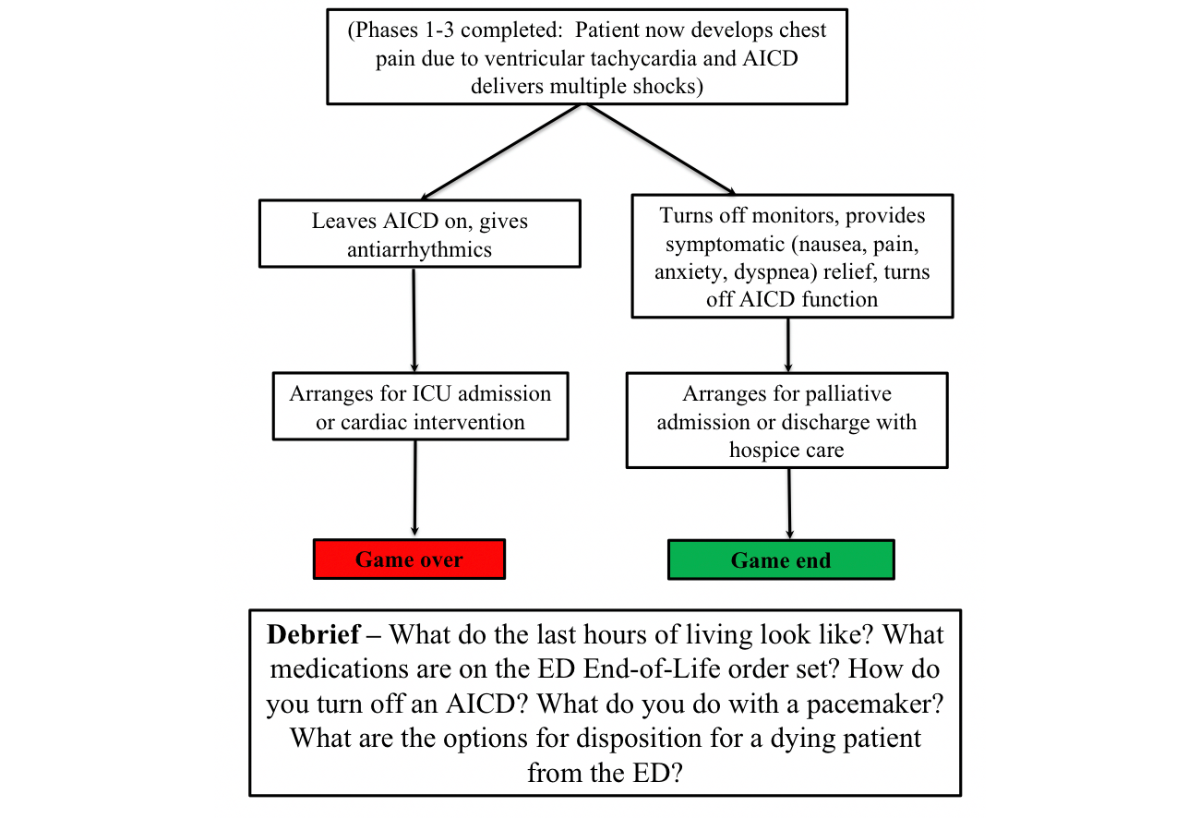
Level 4—Critical action: provide comfort-focused care. AICD: automatic implantable cardioverter defibrillator; ED: emergency department; ICU: intensive care unit.

At the beginning of level 4, comfort-focused care has been clearly established and the patient is dying (Figure 4). The patient’s cardiac rhythm changes and they develop symptomatic ventricular tachycardia. The patient (portrayed by a live actor) is distressed by the rhythm change and experiences crushing chest pain, which results in the AICD firing. The critical action during this last level is to provide appropriate pharmacological, social, and emotional treatment for a patient who is dying. If the learner continues down an advanced cardiac life support pathway, the level is failed (game *over*). If the learners provide symptomatic relief using the comfort care order set and disabling the AICD with a magnet, the level is achieved, and the game is completed. During the debrief, the faculty educates about pacemakers and AICD management at the end of life and discuss disposition planning for the patient (home vs hospice or palliative care in hospital).

### Ethical Considerations

This study was deemed exempt by the Mayo Clinic institutional review board in April. Data were collected in an anonymized fashion.

### Setting

The gaming simulation was developed and deployed for the Mayo Clinic EM Residency at the Mayo Clinic Multidisciplinary Simulation Center in Rochester, Minnesota, United States. A single-session high-fidelity simulation-based intervention was produced and administered 4 times to provide exposure to available cohort members (postgraduate EM residents year 1 through 3) in mid-April 2021. Each session lasted 4 hours.

### Participants

EM residents attend up to six 4-hour immersive educational simulation sessions per academic year, if their clinical schedule allows. The educational intervention was conducted in April. Two of the facilitators were core simulation faculty and had previously established longitudinal teaching relationships with the trainees. The remaining facilitators participated as palliative content experts and had no prior simulation teaching context with the learners.

### Measures

The Simulation Effectiveness Tool—Modified (SET-M) survey tool is a validated method developed to assess learners’ perceptions of how well the simulation instruction met their learning needs in relation to the specific topic [18]. The psychometric quality of the instrument has been reported (Cronbach α ranged between .729 and .874) and externally validated as a valid, accurate, and reliable educational tool [19,20]. The questions in this survey focus on four domains: (1) prebriefing, (2) debriefing, (3) learning, and (4) confidence. Each domain is composed of several declarative statements about the perceptions of the simulation instrument. The SET-M survey was obtained on the internet and manually entered into Research Electronic Data Capture (REDCap; Vanderbilt University), a secure web application for managing web-based surveys and databases. The survey was then electronically distributed via email to participants the day after completion of the gaming simulation session. Data were analyzed after a waiting period of 1 month following survey distribution.

## Results

### Overview

Out of 27 EM residents at Mayo Clinic, 20 (74%) were available to participate in the educational gaming simulation sessions based on clinical rotations. Resident demographic information was supplied by the EM residency director in order to allow those surveyed to remain anonymous. Of the participating resident physicians, 70% (14/20) of them were White and 70% (14/20) of them were female. Learner groups consisted of 4 to 6 EM resident physicians (a mixture of postgraduate years 1-3), in addition to an emergency nurse and respiratory therapist. The residents and nurses were blinded to the topic of the gaming simulation but were prebriefed and instructed on LDR gameplay format before starting the module, with attention given to its task-oriented and recursive nature. Rotating teams of 3 learners consisting of 1 senior EM resident, 1 junior EM resident, and 1 emergency nurse participated in each level.

### Survey Results

Eighty percent (16/20) of residents completed the SET-M survey and nearly 100% (20/20) of them strongly or somewhat agree that this gaming simulation format was effective in improving skills and confidence caring for a patient at the end of life in the following dimensions: (1) better prepared to respond to changes in condition, (2) more confident in assessment skills, (3) teaching patients, (4) reporting to the health care team, (5) empowered to make clinical decisions, and (6) able to prioritize care and interventions. The results of SET-M by domains: prebriefing, debriefing, learning, and confidence are displayed in Table 1.

**Table 1:** Results of the Simulation Effectiveness Tool—Modified survey.

				Strongly agree		Somewhat agree		Disagree		No response
**Prebriefing**										
	Increased my confidence			7		5		1		3
	Beneficial to my learning			—^a^		—		—		—
**Debriefing**										
	Valuable in helping me improve my clinical judgment			14		2		0		—
	Provided opportunities to self-reflect on performance			15		1		0		—
	Constructive evaluation of the simulation			15		1		0		—
	Debriefing contributed to learning			15		0		0		1
**Learning**										
	Better prepared to respond to changes in patient condition			15		1		0		
	Better understanding of pathophysiology			9		7		0		
	More confident of assessment skills			12		4		0		
	Empowered to make clinical decisions			12		2		0		2
	Better understand medications			11		5		0		
**Confidence**										
	**More confident**			—		—		—		—
		Using evidence-based practice	10		6		0		—	
		Reporting information to medical team	10		6		0		—	
		Providing interventions fostering safety	10		5		0		1	
		Ability to prioritize care and interventions	14		2		0		—	
		Teaching patients about illness	13		3		0		—	
		Communicating with patient	14		1		1		—	

^a^Not available.

### Results of the Simulation Effectiveness Tool—Modified Survey

Overall, most residents answered that a short discussion prior to the start of the gaming simulation (prebriefing) was beneficial for learning. In the debriefing stage, 100% (16/16) of the resident learners felt that debriefing contributed to learning and was valuable in helping them improve clinical judgment, provided opportunities to self-reflect on performance and was constructive.

Regarding the learning component, this gaming simulation strongly emphasized communication skills used to conduct an informed goals-of-care discussion and integrated teaching on pharmacologic and nonpharmacologic symptom management at the end of life and anticipatory interventions such as deactivation of an implantable defibrillator. There were 94% (15/16) of residents who strongly agree and 6% (1/16) of those who somewhat agree that they felt better prepared to respond to changes in patients’ condition; 56% (9/16) of them strongly agree and 44% (7/16) of them somewhat agree that they had a better understanding of pathophysiology; 75% (12/16) of them strongly agree and 25% (4/16) of them somewhat agree that they were more confident in their assessment skills; 75% (12/16) of them strongly agree and 13% (2/16) of them somewhat agree they feel empowered to make clinical decisions; and 69% (11/16) of them strongly agree and 31% (5/16) of them somewhat agree they had a better understanding of the medications and therapies after the gaming simulation.

Regarding confidence, most residents (14/16, 88%) strongly agreed that they were more confident communicating with their patients and felt more confident prioritizing interventions including understanding patients’ goals of care through a surrogate and ACP documents and providing care which aligned with values of comfort-focused care. Residents also felt more confident educating the patient and care partners about their illness and prognosis (which for this patient was death).

Learner comments about the simulated clinical experience highlighted the positive impact the gaming simulation had on their ability to have an informed goals-of-care discussion. Thirteen of 16 (81%) respondents provided comments indicating the gaming simulation was valuable (Multimedia Appendix 1). One resident specifically commented on the LDR methodology used.

## Discussion

### Overview

The results of this study suggest the LDR palliative gaming simulation was perceived as an effective tool to deliver critical concepts related to end-of-life care.

### Principal Findings

Previously, it was unknown whether LDR, which was developed with the explicit aim to teach procedural and intervention-based resuscitation, would be applicable in a palliative end-of-life care situation. Sunga et al [12] evaluated the effectiveness of LDR and found the format achieved level 1 using the Kirkpatrick Model for evaluation of training methods, indicating learners found the gaming simulation format engaging and relevant. This was thought to be due to multiple factors such as gamification qualities, inherently fatalistic approach alleviating learner self-doubt, and opportunity for stress inoculation and deliberate practice [12]. In contrast, the patient in this scenario was dying and the case required a shift to patient-centered communication and symptom management instead of a reflexive disease diagnosis and treatment paradigm.

### Strengths

The LDR palliative module was similarly well-received by EM residents despite its nonresuscitation care focus and a high potential for failure on the first attempt. For example, during these scenarios, many of the resident groups failed to achieve each level on the initial attempt, but this was not reflected in the SET-M survey as a negative perception of the gaming simulation instrument. On the contrary, the results of the survey assessing the debriefings were universally positive. Debriefing is where the deliberate practice component of education about communication occurred. The gaming simulation itself became an opportunity to reflect on the behaviors and actions and plan for a possible remedial action. Furthermore, the LDR model has applications in the 4 Kolb stages of learning, which describe an integrated process, with each stage being mutually supportive of and feeding into the next [21]. Effective learning occurs when the learner can execute all four stages of the model: (1) participation on a critical action–bounded scenario (concrete experience), (2) teacher-assisted self-reflection and behavior-specific feedback (reflective observation), (3) explication of critical actions and its consequences as a failure or success (abstract conceptualization), and (4) rekindling of the scenario with recently acquired new skills (active experimentation) [21].

### Comparison With Prior Studies

The skills gained through this gaming simulation focused on patient-provider communication as well as pharmacologic and nonpharmacologic symptom management of dyspnea, pain, nausea, agitation at the end of life, and anticipatory interventions such as deactivation of an implantable defibrillator. We used SET-M, a reliable and validated tool [18,22] to evaluate residents’ perception of how effective the gaming simulation was toward meeting their learning needs. This tool has previously been used to evaluate end-of-life care among pediatric intensive care unit nurses, tele-simulation for medical student education, intubation during COVID-19, and in situ simulations for safety [23-26]. Through this tool, we found that resident physician levels of learning, communication, and satisfaction with debriefings were excellent. Residents reported being more confident communicating with their patient and felt more confident prioritizing interventions including understanding patients’ goals of care and providing care which aligned with the values of the patient. Residents also felt more confident educating the patient and care partners about their illness and empowered to make medical decisions.

To obtain procedural competency, EM residents are taught a stepwise approach and have many opportunities to practice the procedure over time. EM trainees, in general, receive little education regarding care of the dying patient [4-6]. Like mastery of critical procedures, EM residents need education and practice mastering the skills needed to efficiently discuss and determine goals of care for a patient who is dying in the ED. This requires identifying and interpreting relevant ACP documents, confirming these wishes with the patient, and making a tailored management recommendation based on the clinical scenario and the patient's previous wishes. This gaming simulation incorporated these tasks as critical actions with thorough debriefs. Based on the feedback from this gaming simulation, it appears that gaming simulation in general may be an effective way to teach EM residents valuable communication skills for patients at the end of life. This also aligns with previous studies that have demonstrated that simulation can be effective in palliative care education in the ED [9].

### Limitations

First, 4 residents did not complete the SET-M survey even after sending multiple requests to complete it, and it is possible those residents were dissatisfied with the gaming simulation including the content or the novel methodology used for delivery through LDR. Second, the results of this study may not necessarily be applicable to a larger setting or to learners at other levels of training such as medical students, attending physicians, or non-EM trainees. Efficacy of LDR with regard to Kirkpatrick Model levels 2-4 (knowledge acquisition, behavior change, and outcomes) was not evaluated with this project. Third, the success of this gaming simulation demands expertise in the designers and facilitators in end-of-life care and communication. There is no clear validated approach to clarifying goals of care and code status in the imminently dying patient, which makes evaluation of the skills challenging. The LIIFE framework discussed and taught during this gaming simulation was created by one of the ED faculty and has not been studied or previously published at the time that this paper was written.

While most of the residents felt that this gaming simulation was effective in improving skills and confidence caring for a patient at the end of life, not all trainees learn best in simulated scenarios [25]. One trainee felt quite uncomfortable during the gaming simulation session and mentioned performing in front of their peers was uncomfortable based on their written feedback. Fourth, the outcomes measured come from a single survey that was distributed after curricular implementation, which makes it susceptible to recall bias and lack of a control or comparison group. The population studied was also quite small and fairly homogeneous at a single institution, which limits the generalizability of the study’s findings.

Finally, 1 question in the SET-M survey was accidentally excluded from the formal survey completed. The question asked if residents had the opportunity to practice clinical decision-making skills. We believe the absence of this question does not significantly impair the results given the recursive nature of the gaming simulation format.

### Conclusions

The LDR palliative gaming simulation module was perceived by residents to be effective at improving learning and confidence regarding end-of-life care and communication. Further work is needed to determine whether LDR as a tool for palliative education can improve the retention of learned concepts, affect future performance, transfer to non-EM settings, and contribute to positive patient-based outcomes.

## Appendix

Multimedia Appendix 1 Resident physician participant comments.

## Abbreviations

ACP: advance care planning
AICD: automatic implantable cardioverter defibrillator
ED: emergency department
EM: emergency medicine
EMS: emergency medical services
LDR: LIVE. DIE. REPEAT
POLST: provider order for life-sustaining treatment
REDCap: Research Electronic Data Capture
SET-M: Simulation Effectiveness Tool—Modified
STROBE: Strengthening the Reporting of Observational Studies in Epidemiology

## Data Availability

All data generated or analyzed during this study are included in this paper (and its supplementary information files).
