# Supplementary material for: Teaching Palliative Care to Emergency Medicine Residents Using Gamified Deliberate Practice-Based Simulation: Palliative Gaming Simulation Study
Source: JMIR Med Educ. 2023 Aug 16;9:e43710. doi: 10.2196/43710 (PMC10468704; doi:10.2196/43710)
Supplement: Multimedia Appendix 1 [file mededu_v9i1e43710_app1.docx]

| **Resident Physician** | **Comments** |
| --- | --- |
| 1 | We didn’t perform a pre-briefing so I wasn’t sure how to answer these questions. Excellent SIM, highly valuable and applicable to personal and professional life. |
| 2 | This simulation was incredibly useful to help learn strategies to facilitate goals of care discussion and end of life care. It has significantly improved my confidence in addressing end of life discussions and helping patients and families through end of life discussion and care. |
| 3 | Absolutely fantastic session. So well organized, loved the live die repeat format, LOVED the special guests and their contributions and the entire discussion was so thoughtful and supportive. Absolutely loved it. |
| 4 | The framework provided for addressing palliative care in the ED was helpful. Some of the phrasing like “best care possible” was also helpful. |
| 5 | This was an outstanding simulation of a vital and commonly encountered but uncommonly taught scenario. Palliative care skills are rarely tested on paper but are among the most valuable skills to improve for the benefit of our patients, our patients’ families and the teams we lead and ourselves. |
| 6 | It was such a unique opportunity to lean that we are so grateful to have had during residency. It is training that is crucial to our curriculum! |
| 7 | What an incredible experience overall. I never anticipated learning so much about palliative care in one day. You definitely sparked an interest in me in something that I previously hadn’t thought much about. Thank you all for taking the time to put this together. |
| 8 | Very effective to increase my skills to have meaningful goals of care discussion. |
| 9 | It was fantastic. |
| 10 | Thank you for focusing on this often-neglected part of patient care. Just the next shift after my SIM I had 2 opportunities to put what I learned into action, to the patients benefit. It makes me think of how many opportunities I may have missed before the SIM gave me the confidence, tools, and scripting for these end of life decisions. |
| 11 | There was no pre-briefing that prepared me for this specific simulation case. The debriefing was extremely helpful and necessary in order to grow as a physician. Thank you. |
| 12 | It was an excellent way to hone in on the difficulties of end of life care in the emergency department. Far better than any lecture could have ever been. |
| 13 | Excellent session. |
| 14 | There was an instance in which I made an effort to communicate the risks of intubation…I got feedback from a facilitator during debriefing that their hopes were not to paint such negative light for intubation…This opened the door to more feedback from multiple peers…I am again uncomfortable with simulations in general…Feedback is part of medicine… |

Resident Physician Participant Comments
